# *Mycobacterium tuberculosis* Complex in Remains of 18th–19th Century Slaves, Brazil

**DOI:** 10.3201/eid1905.120193

**Published:** 2013-05

**Authors:** Lauren H. Jaeger, Sheila M.F.M. de Souza, Ondemar F. Dias, Alena M. Iñiguez

**Affiliations:** Fundação Oswaldo Cruz, Rio de Janeiro, Brazil (L.H. Jaeger, S.M.F.M. de Souza, A.M. Iñiguez);; Instituto de Arqueologia Brasileira, Belford Roxo, Brazil (O.F. Dias).

**Keywords:** tuberculosis, TB, Mycobacterium tuberculosis complex, MTC, bacteria, slavery, Brazil, Rio de Janeiro, paleogenetic analysis, ancient DNA, aDNA, mitochondrial DNA, mtDNA, tuberculosis and other mycobacteria

**To the Editor:** Nineteenth century Rio de Janeiro, Brazil, was marked by increased illness and deaths from tuberculosis (TB). By the twentieth century, it was still believed that most TB cases in the Americas originated from Europe; the “virgin soil” hypothesis for African (*1*) and Amerindian populations was accepted. However, modern and archeological DNA evidence confirms the wide distribution of *Mycobacterium tuberculosis* complex (MTC) and TB in the Old and New Worlds. 

Rio de Janeiro was a main entry port for millions of Africans captured for the slave trade. Pretos Novos (New Blacks) Cemetery (PNC; 1769–1830) was created in Rio de Janeiro as a burial ground for the many slaves who died at market. Comingled bone fragments (≈5,000) from ≈30 persons were recovered at PNC; most bones were broken and had been exposed to fire (*2*,*3*). Bioanthropological analysis determined most of the bones were from men 18–25 years of age (*2*); none had lesion consistent with TB.

Femurs from 16 persons were surveyed for *M. tuberculosis* ancient DNA (aDNA). The thick shafts of femur offered a preserved condition for molecular analysis, and the bones could be individualized, avoiding duplication of samples. Paleogenetic investigation guidelines were followed. Sample preparation, aDNA extraction, and PCR were performed at the Paleogenetics Unit (Oswaldo Cruz Foundation, Rio de Janeiro) an isolated environment exclusively dedicated to aDNA research.

Before removal of the bone surface, samples were decontaminated by ultraviolet light (15 min/all sides), frozen in liquid nitrogen, and subjected to manual trituration. Bone powder (≈200 mg) was then incubated with digestion buffer (56°C, 48–72 h) as described (*4*). aDNA hybridizations with MTC probes were conducted as described (*4*). By using 2 segments of mitochondrial DNA (mtDNA) hypervariable segment I (HVS-I) target, we determined the ancestry of the humans from whom the bones were derived (*4*). To control for recent contamination, we compared the HVS-I sequences with those in GenBank and also in a database for the laboratory staff.

Using the hybridization assay with insertion sequence (IS) 6110 target, we detected MTC aDNA in bones from 4/16 persons (samples PN1, PN8, PN13, PN15); 3 of these samples (PN1, PN8, PN13) were also positive for IS1081 target, confirming MTC infection (Table). HVS-I target was retrieved from 3 samples (PN6, PN14, PN15), which enabled determination of the human mtDNA haplotypes (L3e2, L3d1, L1c2, respectively) (Table). The haplotypes showed that the 3 persons were of African descent (GenBank accession nos. JQ639893–Q639895). Our findings are consistent with those from studies based on current African populations, which show that haplotype L1c is restricted to central Africa (*5*) and L3d and L3e are most frequently found in western and central Africa, respectively (*6*).

**Table:** Results of genetic analyses of *Mycobacterium tuberculosis* complex hybridization and human mtDNA haplotypes from human bone samples collected from Pretos Novos Cemetery, Rio de Janeiro, Brazil*

Sample	TB hybridization			Nucleotide position of mtDNA hypervariable segment I†												mtDNA haplotype
	IS6110	IS1081		129	145	148	172	187	189	213	223	278	311	319	320	
CRS	NA	NA		G	G	C	T	C	C	G	C	C	T	G	C	H
PN1	+	+														
PN2	−	−														
PN3	−	−														
PN4	−	−														
PN5	−	−														
PN6	−	−		G	G	C	T	C	T	G	T	C	C	G	T	L3e2
PN7	−	−														
PN8	+	+														
PN9	−	−														
PN10	−	−														
PN11	−	−														
PN12	−	−														
PN13	+	+														
PN14	−	−		G	G	C	T	C	T	G	T	C	T	A	C	L3d1
PN15	+	−		A	A	C	T	T	C	A	T					L1c2
PN16	−	−														

*The cemetery was used as a burial ground for African slaves who died in slave markets during 1769–1830. Blank cells mean target could not be retrieved. mtDNA, mitochondrial DNA; TB, tuberculosis; IS, insertion sequence; CRS, Cambridge Reference Sequence (accession no. AB055387); NA, not applicable; PN, bone samples from humans buried in Pretos Novos Cemetery; Abs, absence of nucleotide. †The prefix 16 has been omitted from the nucleotide numbers.

Historic data (*3*) showed that 95% of persons buried in PNC were New Blacks, meaning they were born in Africa and died just after arriving in America. Our mtDNA results confirm historic and genetic records that indicate a large percentage of persons brought to Brazil as slaves originated from western–central and western Africa. This makes the PNC samples unique for the paleogenetic purpose of this investigation.

The endemicity of TB in Rio de Janeiro during the colonial period was confirmed by Jaeger and colleagues, who demonstrated MTC infection in 56.6% of persons with European ancestry buried at Nossa Senhora do Carmo Church (*4*). The difference in the frequency of MTC found in the remains of slaves buried in PNC and of Europeans buried at Nossa Senhora do Carmo Church may be explained by the types of samples analyzed and the epidemiologic conditions of both groups. The cremation of corpses at PNC may also partly explain the difference. The finding of MTC aDNA in the remains of 25% of persons buried at PNC could be an underestimation of infection.

Our finding that some of the slaves buried in PNC had TB infection when they arrived in Brazil is in agreement with previous findings of the differential distribution of TB and with a tuberculin survey on the African continent, supporting the hypothesis of native African TB (*7*,*8*). Therefore, the hypothesis of Africa as virgin soil for TB (*1*,*9*) cannot be easily supported. The incidence of TB among the slaves/Blacks in Rio de Janeiro was less than expected given their social and sanitary conditions (*10*), especially in a TB-endemic situation (*4*). Previous exposure to MTC might explain their apparent relative resistance.

Other evidence showing African contact with Europeans before the sixteenth century, supports the existence of TB in Africa (*8*), and TB was prevalent in urbanized centers along coastal areas of western Africa (*7*,*8*). Although some of those cases were probably the result of European contact, it is not possible to exclude that some were caused by TB native to Africa. We can affirm that persons buried in PNC, who were transported to Brazil as slaves from Africa, brought TB infection with them; whether the infection was caused by European TB endemic to Africa or by TB native to Africa is not known.
